# Hybrid integrated biological–solid-state system powered with adenosine triphosphate

**DOI:** 10.1038/ncomms10070

**Published:** 2015-12-07

**Authors:** Jared M. Roseman, Jianxun Lin, Siddharth Ramakrishnan, Jacob K. Rosenstein, Kenneth L. Shepard

**Affiliations:** 1 Department of Electrical Engineering, Columbia University, New York, New York 10027, USA; 2 Department of Biology, Neuroscience Program, University of Puget Sound, Tacoma, Washington 98416, USA; 3 School of Engineering, Brown University, Providence, Rhode Island 02912, USA

## Abstract

There is enormous potential in combining the capabilities of the biological and the solid state to create hybrid engineered systems. While there have been recent efforts to harness power from naturally occurring potentials in living systems in plants and animals to power complementary metal-oxide-semiconductor integrated circuits, here we report the first successful effort to isolate the energetics of an electrogenic ion pump in an engineered *in vitro* environment to power such an artificial system. An integrated circuit is powered by adenosine triphosphate through the action of Na^+^/K^+^ adenosine triphosphatases in an integrated *in vitro* lipid bilayer membrane. The ion pumps (active in the membrane at numbers exceeding 2 × 10^6^ mm^−2^) are able to sustain a short-circuit current of 32.6 pA mm^−2^ and an open-circuit voltage of 78 mV, providing for a maximum power transfer of 1.27 pW mm^−2^ from a single bilayer. Two series-stacked bilayers provide a voltage sufficient to operate an integrated circuit with a conversion efficiency of chemical to electrical energy of 14.9%.

## Abstract (web summary)

There is enormous potential in combining the capabilities of the biological and the solid-state to create hybrid engineered systems. Here, the authors develop a technique to incorporate and activate ATPases in *in vitro* membranes to produce energy-harvestable currents to power an integrated circuit.

## Graphical abstract

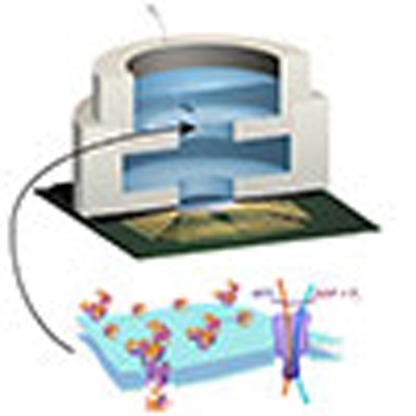

The energetics of living systems are based on electrochemical membrane potentials that are present in cell plasma membranes, the inner membrane of mitochondria, or the thylakoid membrane of chloroplasts^1^. In the latter two cases, the specific membrane potential is known as the proton-motive force and is used by proton adenosine triphosphate (ATP) synthases to produce ATP. In the former case, Na^+^/K^+^-ATPases hydrolyse ATP to maintain the resting potential in most cells.

While there have been recent efforts to harness power from some naturally occurring potentials in living systems that are the result of ion pump action both in plants^2^ and animals^3^,^4^ to power complementary metal-oxide semiconductor (CMOS) integrated circuits (ICs), this work is the first successful effort to isolate the energetics of an electrogenic ion pump in an engineered *in vitro* environment to power such an artificial system. Prior efforts to harness power from *in vitro* membrane systems incorporating ion-pumping ATPases^5^,^6^,^7^,^8^,^9^ and light-activated bacteriorhodopsin^9^,^10^,^11^ have been limited by difficulty in incorporating these proteins in sufficient quantity to attain measurable current and in achieving sufficiently large membrane resistances to harness these currents. Both problems are solved in this effort to power an IC from ATP in an *in vitro* environment. The resulting measurements provide new insight into a generalized circuit model, which allows us to determine the conditions to maximize the efficiency of harnessing chemical energy through the action of electrogenic ion pumps.

## Results

### ATP-powered IC

**Figure 1:**
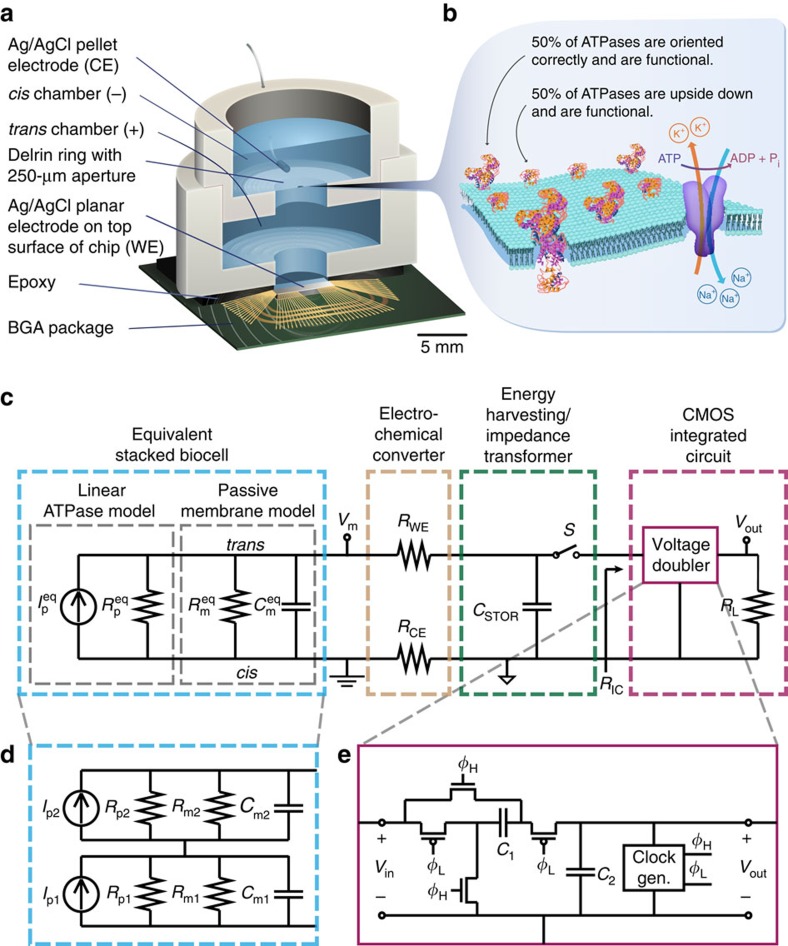
Fully hybrid biological–solid-state system. (**a**) Illustration depicting biocell attached to CMOS integrated circuit. (**b**) Illustration of membrane in pore containing sodium–potassium pumps. (**c**) Circuit model of equivalent stacked membranes, 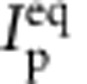
=2.1 pA, 
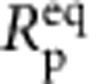
=98.6 GΩ, 
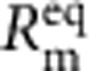
=575 GΩ and 
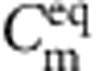
=75 pF, Ag/AgCl electrode equivalent resistance *R*_WE_+*R*_CE_<20 kΩ, energy-harvesting capacitor *C*_STOR_=100 nF combined with switch as an impedance transformation network (only one switch necessary due to small duty cycle), and CMOS IC voltage doubler and resistor representing digital switching load. *R*_L_ represents the four independent ring oscillator loads. (**d**) Equivalent circuit detail of stacked biocell. (**e**) Switched-capacitor voltage doubler circuit schematic.

Figure 1a shows the complete hybrid integrated system, consisting of a CMOS IC packaged with an ATP-harvesting ‘biocell'. The biocell consists of two series-stacked ATPase bearing suspended lipid bilayers with a fluid chamber directly on top of the IC. Series stacking of two membranes is necessary to provide the required start-up voltage for IC and eliminates the need for an external energy source, which is typically required to start circuits from low-voltage supplies^2^,^3^. As shown in Fig. 1c, a matching network in the form of a switched capacitor allows the load resistance of the IC to be matched to that presented by the biocell. In principle, the switch S can be implicit. The biocell charges *C*_STOR_ until the self start-up voltage, *V*_start_, is reached. The chip then operates until the biocell voltage drops below the minimum supply voltage for operation, *V*_min_. Active current draw from the IC stops at this point, allowing the charge to build up again on *C*_STOR_. In our case, however, the IC leakage current exceeds 13.5 nA at *V*_start_, more than can be provided by the biocell. As a result, an explicit transistor switch and comparator (outside of the IC) are used for this function in the experimental results presented here, which are not powered by the biocell and not included in energy efficiency calculations (see Supplementary Discussion for additional details). The energy from the biocell is used to operate a voltage converter (voltage doubler) and some simple inverter-based ring oscillators in the IC, which receive power from no other sources.

**Figure 2:**
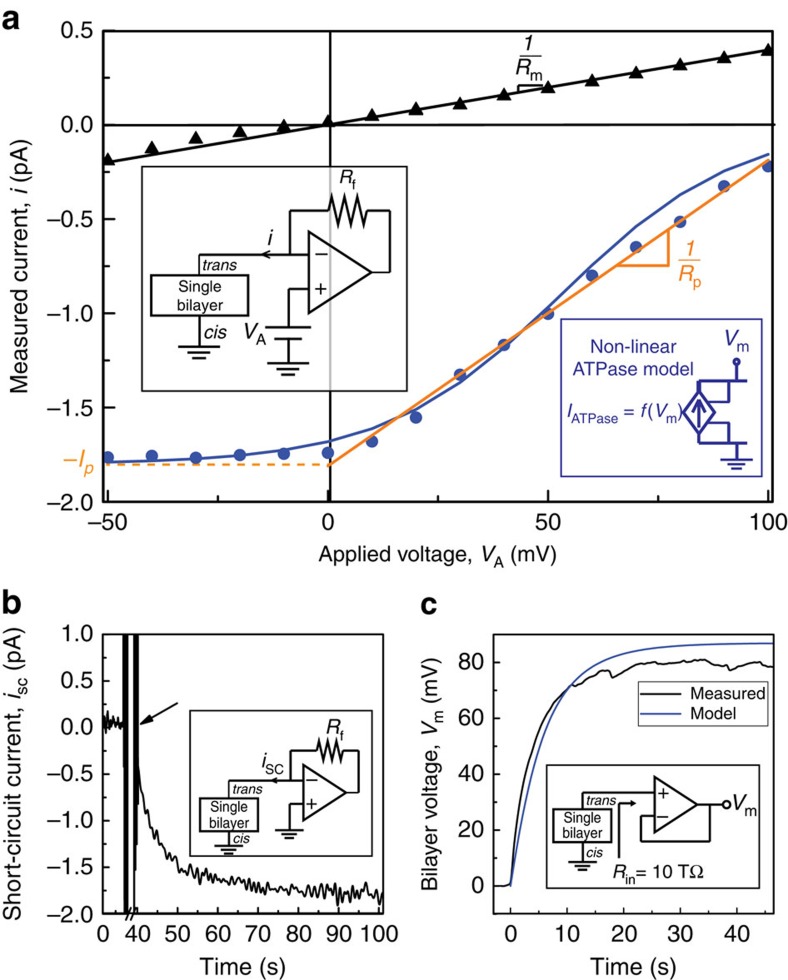
Single-cell biocell characterization. (**a**) *I*–*V* sweeps of biocell before (▴) and after (●) addition of ATP (3 mM final concentration). Voltages are stepped for this measurement. Bilayer current is the average of the last 5 s of a 10-s step (after charging currents have decayed away). Bilayer capacitance *C*_m_=153 pF (consistent with DOPC bilayers^12^,^30^). Pre-ATP data linear fit (black line) slope yield *R*_m_=280 GΩ. Post ATP data fit to a Boltzmann curve, slope=0.02 V (blue line). Post-ATP linear fit (red line) yields *I*_p_=−1.8 pA and *R*_p_=61.6 GΩ, which corresponds to a per-ATP source resistance of 6.16 × 10^15^. The current due to membrane leakage through R_{m} is subtracted in the post-ATP curve. (**b**) Short circuit current magnitude increasing to 1.7 pA after the addition of 3 mM ATP. Transimpedance amplifier gain is 500 GΩ. The headstage filter consists of a three-pole analog low-pass Bessel filter *f*_c_=10 kHz in series with four-pole analog low-pass Bessel filter *f*_c_=100 Hz. Data is post-processed with a low-pass digital filter with *f*_c_=1 Hz. (**c**) Biocell charging its own capacitance to 78 mV. Measured (black line). Simulated using non-linear model fit (blue line).

A silver/silver-chloride (Ag/AgCl) microfabricated thin film on the surface of the chip, and an Ag/AgCl pellet serve as the working (WE) and counter (CE) electrode to convert ions to electrons. Careful attention must be paid to the electrodes in this power-transfer application to ensure that the electrodes are not an uncontrolled galvanic energy source. The lipid (purified 1,2-dioleoyl-*sn*-glycero-3-phosphocholine, DOPC) bilayers are formed in a 250-μm pore^12^ epoxied to a teflon housing (see Methods for details). Purified 5′-sodium potassium adenosine triphosphatase from porcine cerebral cortex is embedded into each bilayer (see Methods for details). Prior to the addition of ATP, the membrane produces no electrical power and has an *R*_m_ of 280 GΩ. A 1.7-pA short-circuit (SC) current (Fig. 2b) through the membrane is observed upon the addition of ATP (final concentration 3 mM) to the *cis* chamber where functional, properly oriented enzymes generate a net electrogenic pump current. To perform these measurements, currents through each membrane of the biocell are measured using a voltage-clamp amplifier (inset of Fig. 2b) with a gain of 500 GΩ with special efforts taken to compensate amplifier leakage currents. Each ATPase transports three Na^+^ ions from the *cis* chamber to the *trans* chamber and two K^+^ ions from the *trans* chamber to the *cis* chamber (a net charge movement of one cation) for every molecule of ATP hydrolysed. At a rate of 100 hydrolysis events per second under zero electrical (SC) bias^13^, this results in an electrogenic current of ∼16 aA. The observed SC current corresponds to about 10^5^ active ATPases in the membrane or a concentration of about 2 × 10^6^ mm^−2^, about 5% of the density of channels occurring naturally in mammalian nerve fibres^14^. It is expected that half of the channels inserted are inactive because they are oriented incorrectly.

### Current–voltage characteristics of the ATPases

Figure 2a shows the complete measured current–voltage (*I*–*V*) characteristic of a single ATPase-bearing membrane in the presence of ATP. The current due to membrane leakage through *R*_m_ is subtracted in the post-ATP curve. The *I*–*V* characteristic fits a Boltzmann sigmoid curve, consistent with sodium–potassium pump currents measured on membrane patches at similar buffer conditions^13^,^15^,^16^. This nonlinear behaviour reflects the fact that the full ATPase transport cycle (three Na^+^ ions from *cis* to *trans* and two K^+^ ions from *trans* to *cis*) time increases (the turn-over rate, *k*_ATP_, decreases) as the membrane potential increases^16^. No effect on pump current is expected from any ion concentration gradients produced by the action of the ATPases (see Supplementary Discussion). Using this Boltzmann fit, we can model the biocell as a nonlinear voltage-controlled current source *I*_ATPase_ (inset Fig. 2a), in which the current produced by this source varies as a function of *V*_m_. In the fourth quadrant, where the cell is producing electrical power, this model can be linearized as a Norton equivalent circuit, consisting of a DC current source (*I*_p_) in parallel with a current-limiting resistor (*R*_p_), which acts to limit the current delivered to the load at increasing bias (*I*_ATPase_∼*I*_p_−*V*_m_/*R*_p_). Figure 2c shows the measured and simulated charging of *C*_m_ for a single membrane (open-circuited voltage). A custom amplifier with input resistance *R*_in_>10 TΩ was required for this measurement (see Electrical Measurement Methods).

### Reconciling operating voltage differences

The electrical characteristics of biological systems and solid-state systems are mismatched in their operating voltages. The minimum operating voltage of solid-state systems is determined by the need for transistors to modulate a Maxwell–Boltzmann (MB) distribution of carriers by several orders of magnitude through the application of a potential that is several multiples of *kT*/*q* (where *k* is Boltzmann's constant, *T* is the temperature in degrees Kelvin and *q* is the elementary charge). Biological systems, while operating under the same MB statistics, have no such constraints for operating ion channels since they are controlled by mechanical (or other conformational) processes rather than through modulation of a potential barrier. To bridge this operating voltage mismatch, the circuit includes a switched-capacitor voltage doubler (Fig. 1d) that is capable of self-startup from voltages as low *V*_start_=145 mV (∼5.5 *kT*/*q*) and can be operated continuously from input voltages from as low as *V*_min_=110 mV (see Supplementary Discussion). In the series-stacked biocell with each membrane having approximately equal resistance and capacitance, 
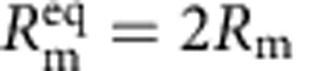
 and 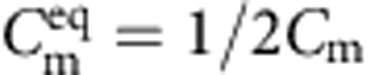. Once the converter has started up, operation can be maintained down to 110 mV, which means that each membrane in the two-stacked configuration can be biased to operate at potentials as low as 55 mV.

### Maximizing the efficiency of harvesting energy from ATP

**Figure 3:**
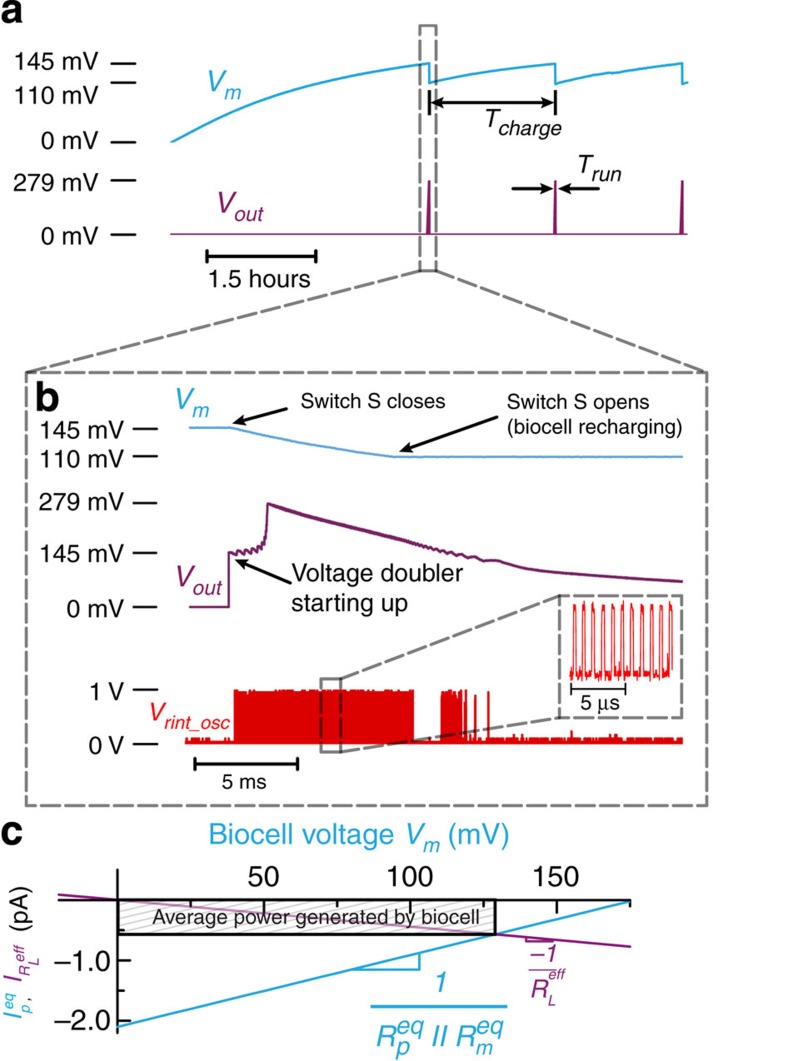
Complete hybrid biological–solid-state system characterization. (**a**) Duty-cycled operation of the stacked biocell and CMOS IC. Shown are the input (*V*_m_) and output (*V*_out_) voltage of the voltage doubler. *T*_charge_=6250, s, *T*_run_=5.5 ms. During *T*_run_, 54.4 nW delivered to the load and 37.9 nW is consumed in running the voltage doubler. (**b**) Expanded waveform showing *V*_m_ and *V*_out_ near a switching transition and one of four ring oscillators. Inset shows the output of ring oscillator digital switching load switching at 1 MHz. (**c**) Biocell current–voltage characteristic and effective load line.

Solid-state systems and biological systems are also mismatched in their operating impedances. In our case, the biocell presents a source impedance, 
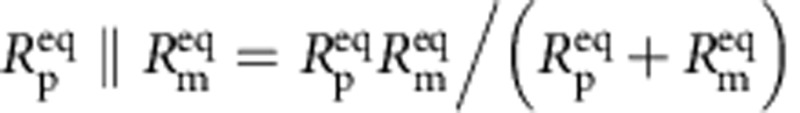
=84.2 GΩ, while the load impedance presented by the complete integrated circuit (including both the voltage converter and ring oscillator loads) is approximately *R*_IC_=200 kΩ. (The load impedance, *R*_L_, of the ring oscillators alone is 305 kΩ.) This mismatch in source and load impedance is manifest in large differences in power densities. In general, integrated circuits, even when operated at the point of minimum energy in subthreshold, consume on the order of 10^−2^ W mm^−2^ (or assuming a typical silicon chip thickness of 250 μm, 4 × 10^−2^ W mm^−3^) (ref. 17). Typical cells, in contrast, consume on the order of 4 × 10^−6^ W mm^−3^ (ref. 18). In our case, a typical active power dissipation for our circuit is 92.3 nW, and the active average harvesting power is 71.4 fW for the biocell. This discrepancy is managed through duty-cycled operation of the IC in which the circuit is largely disabled for long periods of time (*T*_charge_), integrating up the power onto a storage capacitor (*C*_STOR_), which is then expended in a very brief period of activity (*T*_run_), as shown in Fig. 3a.

The overall efficiency of the system in converting chemical energy to the energy consumed in the load ring oscillator (*η*) is given by the product of the conversion efficiency of the voltage doubler (*η*_converter_) and the conversion efficiency of chemical energy to electrical energy in the biocell (*η*_biocell_), *η*=*η*_converter_ × *η*_biocell_. *η*_converter_ is relatively constant over the range of input voltages at ∼59%, as determined by various loading test circuits included in the chip design (Supplementary Figs 1–6). *η*_biocell_, however, varies with transmembrane potential *V*_m_. *η* is the efficiency in transferring power to the power ring oscillator loads from the ATP harvested by biocell.

**Figure 4:**
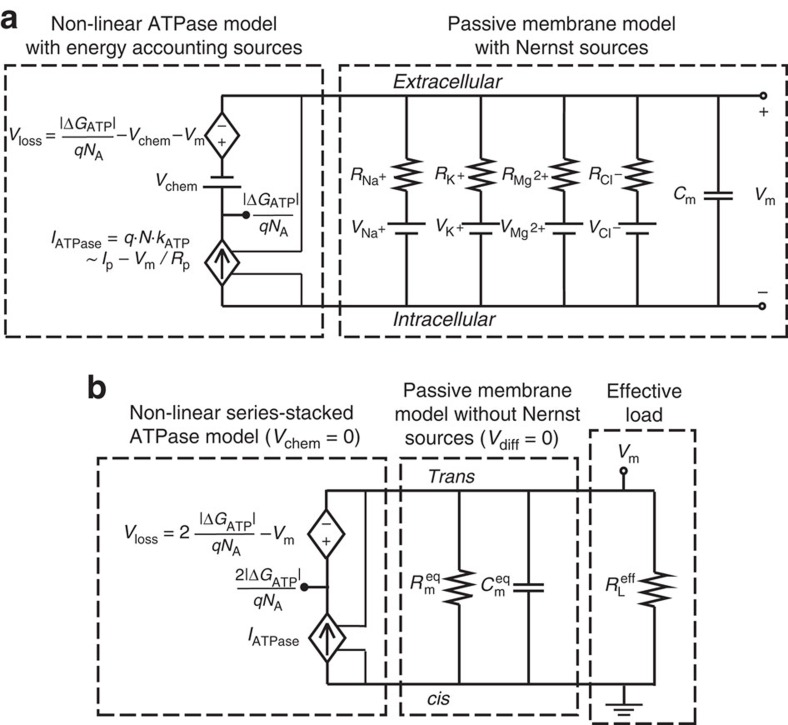
Equivalent circuit models. (**a**) Single-biocell equivalent circuit model with additional voltage sources to account for energy delivered by the pump. (**b**) Stacked membrane equivalent circuit model.

As reflected in the single-membrane circuit model of Fig. 4a, *V*_m_ in cells is the result of two related potentials, both derived from the action of the ion pumps: the potential that develops as a result of ionic concentration gradients of membrane permeable ions (the Nernst, or diffusion, potential, *V*_diff_) and Ohmic potentials (*V*_p_) due to the net electrogenic ionic currents of the ion pumps. These combine to determine the membrane potential, *V*_m_=*V*_diff_+*V*_p_. *V*_diff_ is determined by the superposition of the Thevenin ‘Nernst' sources for each ionic species, 
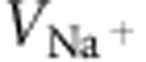
, 
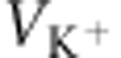
, 
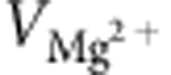
, 
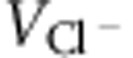
, where 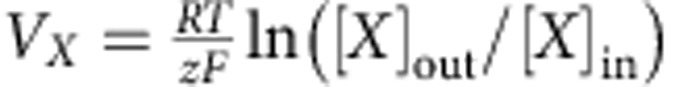, *R* is the gas constant, *z* is the valence of the species, *F* is Faraday's constant and [*X*]_*y*_ is the concentration of species *X* in chamber *y*. A corresponding series resistance *R*_*X*_ models the membrane permeability of those ions present in the system. An equivalent expression for *V*_diff_ is also given by the Goldman–Hodgkin–Katz (GHK) equation. The total membrane resistance *R*_m_ is given by the parallel combination of 
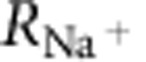
, 
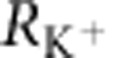
, 
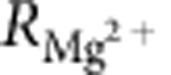
, 
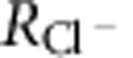
. Using the linear ATPase model (Fig. 1c), the electrogenic component of the transmembrane potential is given by *V*_p_=*I*_p_ (*R*_p_||*R*_m_). Under physiological conditions, *V*_p_ is often negligible compared with *V*_diff_ as a result of the relatively low value of *R*_m_ due to the presence of many transmembrane ion channels.

To first order, the energy made available to the Na^+^/K^+^-ATPase by the hydrolysis of ATP is independent of the chemical or electric potential of the membrane and is given by |Δ*G*_ATP_|/(*qN*_A_), where Δ*G*_ATP_ is the Gibbs free energy change due to the ATP hydrolysis reaction per mole of ATP at given buffer conditions and *N*_A_ is Avogadro's number. Since every charge that passes through *I*_ATPase_ corresponds to a single hydrolysis event, we can use two voltage sources in series with *I*_ATPase_ to independently account for the energy expended by the pumps both in moving charge across the electric potential difference and in moving ions across the chemical potential difference. The dependent voltage source *V*_loss_ in this branch fixes the voltage across *I*_ATPase_, and the total power produced by the pump current source is (|Δ*G*_ATP_|/*N*_A_)(*Nk*_ATP_), which is the product of the energy released per molecule of ATP, the number of active ATPases and the ATP turnover rate. The power dissipated in voltage source *V*_chem_ models the work performed by the ATPases in transporting ions against a concentration gradient. In the case of the Na^+^/K^+^ ATPase, *V*_chem_ is given by 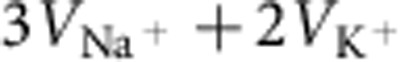. The power dissipated in this source is introduced back into the circuit in the power generated by the Nernst independent voltage sources, 
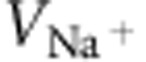
 and 
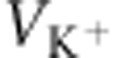
. The power dissipated in the dependent voltage source *V*_loss_ models any additional power not used to perform chemical or electrical work.

In our system, no significant ion concentration gradient exists, *V*_chem_=0, and the expanded membrane model of Fig. 4a becomes the passive membrane model of Fig. 4b. The efficiency of the biocell is given by 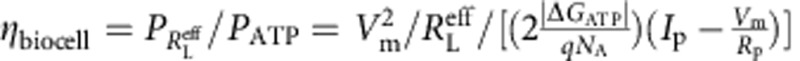 with 
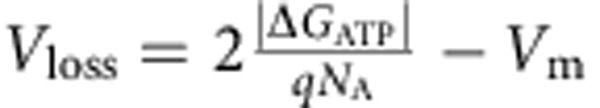
 (the factor of two arises from the series stack, see Supplementary Discussion). The maximum efficiency occurs at 
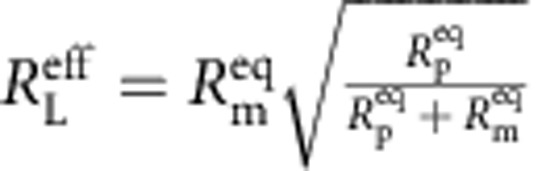
. In our case, 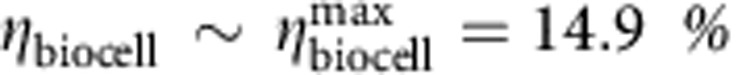 and the overall energy efficiency of the system *η*=*η*_converter_ × *η*_biocell_=8.84%.

Higher membrane potentials can be sustained in systems with electrogenic ion pumps engineered with lower *V*_chem_. For example, proton-pumping ATPases, such as those found in the plasma membrane of the yeast *Schizosaccharomyces pombe*, transport only a single proton per hydrolysed molecule of ATP. Less chemical work is performed (*V*_chem_=14 mV, for ΔpH=0.1) compared with the sodium–potassium pumps. As a result, these pumps can sustain a membrane potential of up to 280 mV^19^,^20^. In our system, with a similar number of active incorporated pumps and *R*_m_ in excess of 575 GΩ due to the absence of other channels, voltages of 227 mV could have been achieved with these pumps.

Living systems use ATP much more efficiently than in the *in vitro* system developed here because *V*_loss_ is considerably smaller in living systems due to the presence of a non-zero *V*_chem_. Under typical physiological conditions, *V*_chem_ is typically on the order of 336.5 mV at 20 °C (ref. 21) with all power lost in *V*_chem_ converted to electrical power by the voltage sources representing the Nernst potentials of the cell.

## Discussion

Integration of ATP-harvesting ion pumps could provide a means to power future CMOS microsystems scaled to the level of individual cells^22^. In molecular diagnostics, the integration of pore-forming proteins such as alpha haemolysin^23^ or MspA porin^24^ with CMOS electronics is already finding application in DNA sequencing^25^. Exploiting the large diversity of function available in transmembrane proteins in these hybrid systems could, for example, lead to highly specific sensing platforms for airborne odorants or soluble molecular entities^26^,^27^. Heavily multiplexed platforms could become high-throughput *in vitro* drug-screening platforms against this diversity of function. In addition, integration of transmembrane proteins with CMOS may become a convenient alternative to fluorescence for coupling to synthetic biological systems^28^.

## Methods

### Teflon cell preparation and lipid bilayer formation

The 250-μm delrin pore is seated in a Teflon cell, washed with 0.1% HCl, 40 mM trisodium phosphate (TSP), rinsed with deionized water, and dried with N_2_. The pore is pretreated with 1 μl of DOPC lipids (Avanti Polar lipids) dissolved in *n*-hexane (5 mg ml^−1^). The hexane is evaporated in a high-vacuum desiccator for 10 min. The Teflon cell is fixed to the chip package (bond wires are protected by donut epoxy encapsulation) and the *cis* and *trans* chambers are filled with experiment buffer (50 mM NaCl, 10 mM KCl, 3 mM MgCl_2_, 10 mM HEPES, pH7.3). A cleaned and equilibrated Ag/AgCl pellet electrode is inserted into the *cis* chamber (ground). The electrode resistance and solution resistance are measured by applying a 500-ms 10-mV voltage step and averaging the current of the last 5 ms of the step response after the charging current transient has decayed away. The electrical resistance is verified to be <20 kΩ (typically 15 kΩ), and the open circuit DC voltage offset between electrodes is confirmed to be <3 mV. If either of these conditions is not met, the electrodes are cleaned as described below.

The lipid bilayer is formed by dipping a plastic pipette tip into a solution of *n*-decane containing DOPC (20 mg ml^−1^), forming an air bubble by depressing the pipette plunger under the surface of the buffer in the *cis* chamber and touching the air bubble to the pore. After 1 min, the bilayer capacitance is extracted by applying a voltage triangle wave and monitoring the resulting current square wave, 
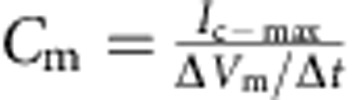
. Bilayers with capacitance <150 pF are reformed by touching another air bubble to the pore. The bilayer resistance is extracted by applying a series of 16 10-s square-wave voltages from −50 to 100 mV in 10 mV increments while monitoring the current. The final 5 s of each step are averaged after charging-current transients have decayed away. The resistance is determined to be the inverse of the slope of a linear fit. Bilayers with resistances <300 GΩ are reformed. We observe that if a successful bilayer cannot be formed within five tries, successive attempts are also likely to be unsuccessful, suggesting that the likelihood of formation of a high resistance stable bilayer is dependent on pore preparation (cleaning and pre-treatment). This is important as the bilayer is reformed many times once the process of ATPase incorporation begins. All measurements are performed in a Faraday cage.

### Electrode preparation

Two identical Ag/AgCl pellet electrodes (Warner Instruments) are used for biocell characterization experiments. Pellet electrodes are shorted together at the wire end for the duration of preparation. The electrodes are immersed in 0.1% HCl for 1 min. The electrodes are rinsed under a DI water stream and immersed in 3% sodium hypochlorite for 30 min. The electrodes are rinsed again under a DI water stream and immersed in experiment buffer (50 mM NaCl, 10 mM KCl, 3 mM MgCl_2_, 10 mM HEPES, pH 7.3) until use. Concentrations employed are very close to physiological conditions, except for the lack of an ion gradient across the membrane. The electrode preparation protocol is repeated if the open-circuit potential offset is found to be above 3 mV. The electrode offsets are verified at the end of each experiment to be <5 mV.

For the IC experiments, the top surface of the IC that is coated with 500 nm of silver by electron-beam evaporation and chloridized with 3% sodium hypochlorite for 30 min serves as the *trans* chamber electrode (working electrode, WE). An Ag/AgCl pellet electrode (counter electrode) is inserted into the *cis* chamber as with the characterization experiments and attached to circuit ground on the board. Cleaning and equilibration of the electrodes are carried out as above; however, the chip is placed in a socket with access to the ‘wire' end of the planar electrode through a pin on the board. A Teflon chamber attached by silicone epoxy (Kwik-cast) to the surface of the chip package serves as a reservoir for the above solutions. It is necessary to momentarily disconnect the ‘wire' ends of the electrodes for the DI water rinse. Electrode offsets exceeding 3 mV are observed more frequently when using the planar electrode; however, we find that this is easily remedied by increasing the chloridization time to 1 h.

### Proteoliposome preparation

Hyperosmotic proteoliposomes to be fused with the planar bilayers are prepared using a modified version of the dual-detergent protocol in ref. 29, which reported that the use of dual detergents yields vesicles with higher transport activity (40–60% of original enzyme activity) than formation using either detergent alone. DOPC dissolved in chloroform (Avanti Polar Lipids) are transferred to a round-bottom flask using a glass syringe (Hamilton). Chloroform is evaporated under high vacuum on a Buchi Rotavap for 5 min. The flask is transferred to a high-vacuum desiccator for a minimum of 2 h to aid in removal of residual solvent. Vesicle buffer containing (575 mM NaCl, 5 mM HEPES, pH 7.3) is added to the flask (final lipid concentration of 4 mg ml^−1^), and the flask is placed on a vortexer (2,500 r.p.m.) for 10 min. Lipid vesicles are mixed with *n*-octyl-β-D-glucopyranoside (*n*-OG; detergent:lipid weight ratio 3:1) and placed on a rotator for 5 min until the vesicle solution become clear. A solution of *n*-decyl-β-D-maltopyranoside dissolved in vesicle buffer is added to purified ATPase (detergent:protein weight ratio 3:1) and the tube is gently rotated for 1 min. Purified ATPase lyophilized powder contains 90% sucrose and 0.4% EDTA. Solubilized vesicles and solubilized ATPase (lipid:protein weight ratio 5:1) are combined in 2-ml centrifuge tubes (total volume of solution 270 μl) and rotated for 1 min. A volume of pre-equilibrated Bio-Beads SM-2 (Bio-Rad) equal to the volume of lipid/protein mixture is added to the tubes and the tubes are placed back on the rotator. After 30 min the tubes are filled to the top with Bio-Beads and rotated for 2 h. Proteoliposomes are removed from Bio-Beads and used immediately.

### Electrical measurement methods

Voltage-clamp (current) measurements are performed using a HEKA EPC10 bench-top patch clamp amplifier. The headstage gain is set to 500 GΩ. Two series programmable analog filters are used in all current measurements.

Filter 1: analog 3-pole Bessel, *f*_c_=10 kHz.

Filter 2: analog 4-pole Bessel *f*_c_=100 Hz.

For *I*–*V* measurements, the last 5 s of a 10-s measurement are averaged.

Patch-clamp amplifiers typically employ large junction gate field-effect transistor input transistors to minimize noise. As a result, the amplifier has large input bias and input offset currents, on the order of a few hundred femtoamps. To compensate for these currents, once a linear fit is applied to the *I*–*V* sweep of a bilayer without ATPases, the measured short-circuit current is subtracted from all data points, such that the curve passes through the origin.

The input resistance, *R*_in_, of the benchtop amplifier is 100 GΩ, which shunts the membrane resistance. This shunt resistance compromises the measurement and instead we use a custom two-stage amplifier using a LMC6041 opamp in non-inverting unity gain feedback followed by an ADA4661 opamp in non-inverting configuration with a gain of 5 V/V. Short time-scale measurements (<1 min) are acquired using an Agilent DSO7054A oscilloscope. Measurements exceeding 1 min are acquired using an Keithley 2182A nanovoltmeter.

## Additional information

**How to cite this article:** Roseman, J. M. *et al.* Hybrid integrated biological–solid-state system powered with adenosine triphosphate. *Nat. Commun.* 6:10070 doi: 10.1038/ncomms10070 (2015).

## Supplementary Material

Supplementary Information Supplementary Figures 1-6, Supplementary Discussion and Supplementary References
