# Supplementary material for: Hybrid integrated biological–solid-state system powered with adenosine triphosphate
Source: Nat Commun. 2015 Dec 7;6:10070. doi: 10.1038/ncomms10070 (PMC4686768; doi:10.1038/ncomms10070)
Supplement: Supplementary Information — Supplementary Figures 1-6, Supplementary Discussion and Supplementary References [file ncomms10070-s1.pdf]

Supplementary Figures

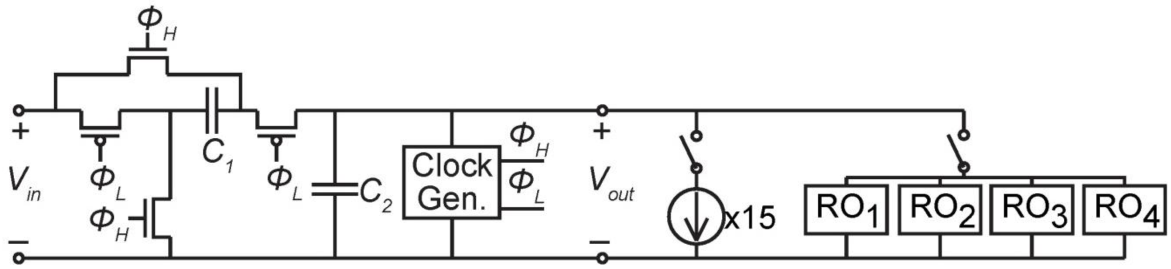

Supplementary Figure 1. Switched-capacitor voltage doubler schematic including 15 calibrated current sources for efficiency characterization and four independent ring oscillator loads.

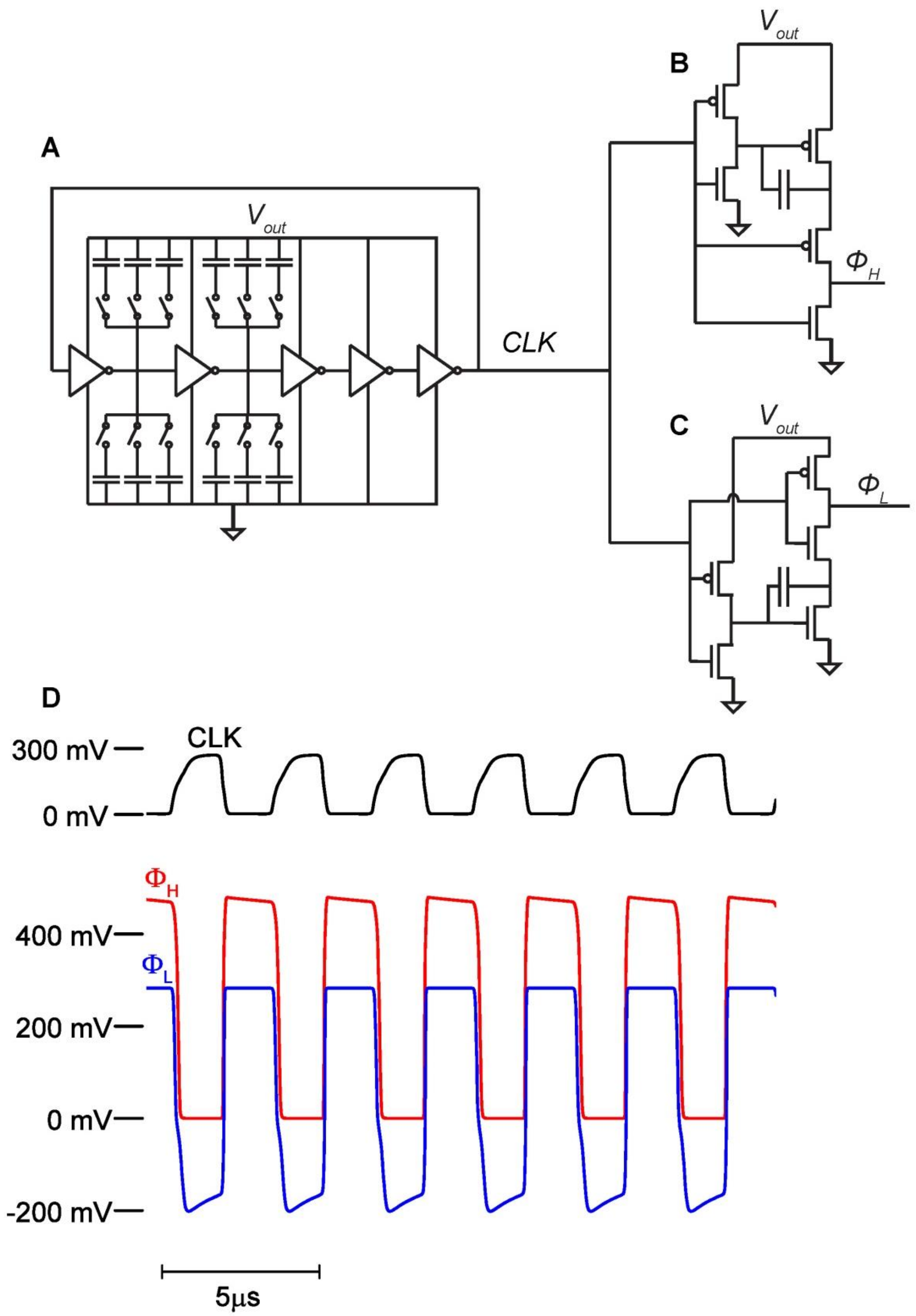

Supplementary Figure 2. Clock generator schematic and simulated output. (**A**) Five-stage ring oscillator with switchable capacitors for frequency tuning. (**B**) Schematic of bootstrap for generating positive clock gate driver Φ_H for NMOS transistors. (**C**) Schematic of bootstrap for generating negative clock gate driver Φ_L for PMOS transistors. Simulated output for V_{out} = 280 mV.

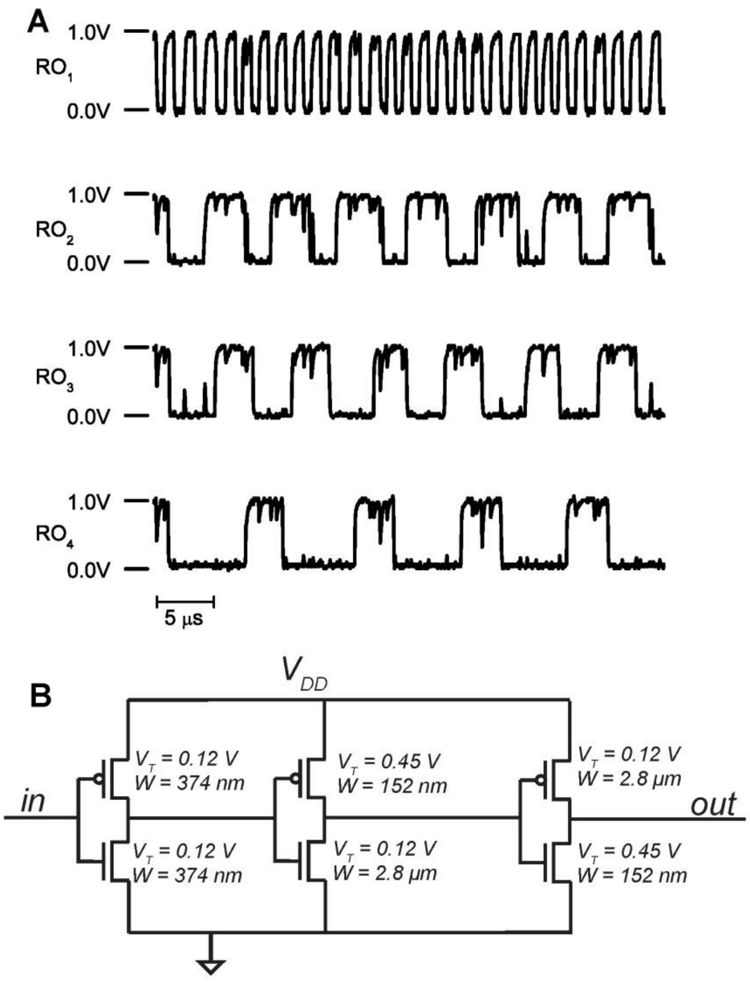

Supplementary Figure 3. Four measured independent ring-oscillator outputs running simultaneously. Ring oscillators are supplied from converter output to simulate digital switching loading of a microprocessor. Low-voltage (<300 mV) to high-voltage (1 V) level shifter used to bring low-voltage signals off the chip. Noise on oscillator outputs attributable to coupling between the oscillators.

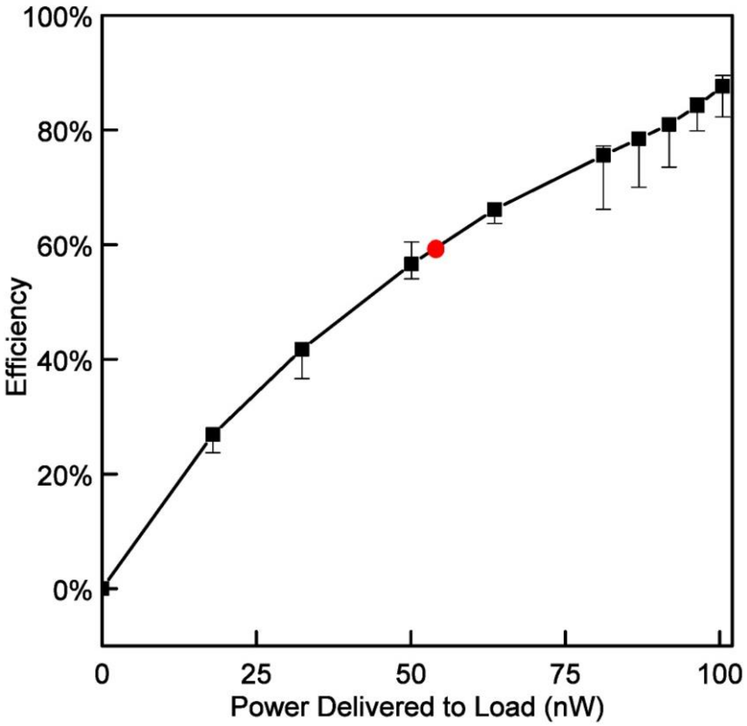

Supplementary Figure 4. Efficiency as a function of load power for V_{in} = 150 mV. Error bars represent minimum and maximum efficiency of four copies of power converter on the same die. The red dot represents the operating point of the converter in our system.

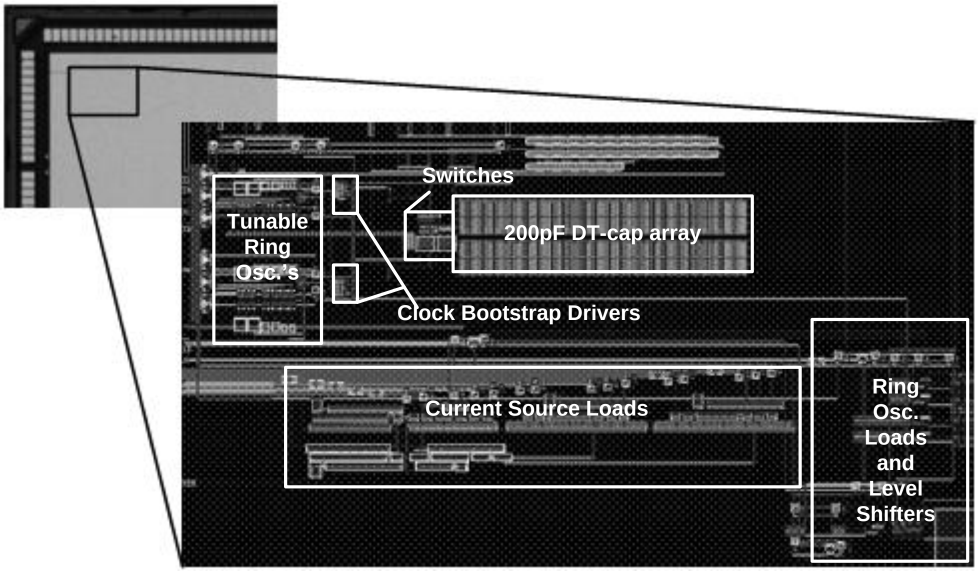

Supplementary Figure 5. Die photo and chip layout. Core converter components (ring oscillators, bootstrap switches and capacitor array) total 0.0015 mm^2. Capacitor array is 66% of total chip area. Total chip area including all auxiliary test circuits is 0.0046 mm^2.

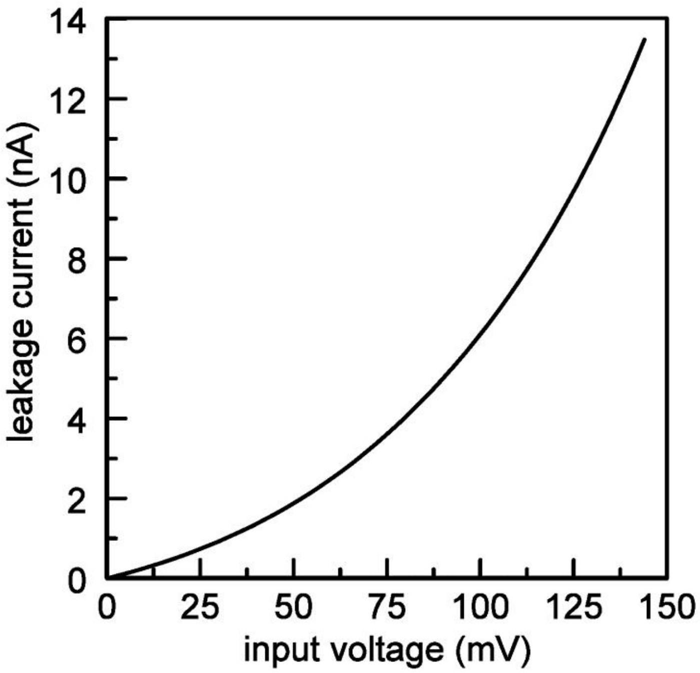

.

Supplementary Figure 6. Leakage current into the chip (including core converter, clocks and loads) at voltages below the startup voltage of 145 mV.

Supplementary Discussion

1. Alternate derivation of ATP efficiency

An alternative method of calculating the efficiency of the biocell in converting the chemical energy released by hydrolysis of ATP to electrical energy transferred to the integrated circuit can be performed by charge counting over a complete charging and discharging cycle. Assuming that the total energy imparted on C_{STOR} during the charging part of the cycle is transferred to the IC during the discharging part of the cycle (valid for $R_{IC} << R_m$), the energy consumed by the load $E_L = \frac{1}{2}C(V_{m-high}^2 - V_{m-low}^2)$. The energy released by ATP hydrolysis is the Gibbs free energy change per molecule of ATP hydrolyzed times the total number of molecules, which is also the total number of charges pumped (which either flow through R_m or serve to charge C_{STOR}). That is, $E_{ATP} = |2\Delta \mathrm{G}_{\mathrm{ATP}}| \times [Q_{R_m} + C_{STOR}\Delta V_m]$, where Q_{R_m} is the total number of charges that flow through R_m, or $\frac{V_{m-avg}}{R_m}T_{charge}$, where $\Delta V_m = V_{m-high} - V_{m-low}$ and $V_{m\text{-}avg}$ is the average voltage during T_{charge}. This yields an efficiency $\eta_{biocell} = \frac{1}{2|2\Delta \mathrm{G}_{\mathrm{ATP}}|}\frac{C_{STOR}(V_{m-high}^2 - V_{m-low}^2)}{\frac{V_{m-avg}}{R_m}T_{charge} + C_{STOR}\Delta V_m}$. For the values given in the main text, this results in $\eta_{biocell}$ = 14.7%, compared with 14.9% using the linear approximation derivation in the main text.

2. Integrated circuit details

The integrated circuit (IC) chip for these studies serves as both the load powered by the biocell and the voltage converter that allows the voltage from the biocell to be increased to one that can effectively power the IC. The chip is implemented in a 45-nm silicon-on-insulator (SOI) CMOS process.

The circuit diagram of the switched-capacitor (SC) voltage doubler is shown in Supplementary Figure 1. Flying capacitor, C_1 = 100 pF, is charged up to the input voltage when the NMOS

transistors are on. When the PMOS transistors are switched on, it is stacked in series with the input and attached to output capacitor, C_2 = 100 pF. Deep-trench capacitors [S1] are employed as the flying and output capacitors in the core converter. These high density capacitors (~0.2µF/mm^2) [S2] have relatively high series resistance and intrinsic time constants of ~10 µs. A set of 15 calibrated current sources are included in the design as loads. Additionally four distinct ring oscillators can be attached to the output as switching digital loads.

To determine $\eta_{converter}$, we must account for power losses in the converter that include both conduction losses (P_{cond}) and switching losses (P_{sw}) [S3]. The losses associated with any additional control circuitry including clocks, have been lumped in with P_{sw}, due to their strong dependence on clock frequency. Maximizing efficiency in the ultra-low power regime necessitates that the design be tuned for a specific target source V_{in}, in this case 150 mV. The design performs well in a small range of inputs around the target source but efficiency quickly drops when V_{in} is more than 200 mV. This limited range is due mostly to the exponential dependence of transistor currents on node voltages in the subthreshold region.

In steady-state operation, the clocks to startup and run the converter are generated by a trimmable five-stage ring oscillator. Transistors with nominal threshold voltages, $|V_{T,lin}|$, of 760 mV are employed. The trimming capacitors, which are necessary to contend with large process variability, as well as selecting f_{clk}, the unloaded switching frequency of the converter, are switched in using pass gates controlled by an external 1.5-V supply brought onto the chip specifically for this purpose. In an autonomous configuration (in which the 1.5-V supply is not available), these could be trimmed with fuses. In a typical SC converter, non-overlapping clocks are used for each of the phases [S3], however simulation reveals that the additional switching logic necessary to support non-overlapping clock generation (>20 nW) outweighs the loss due to short circuit currents (<3nW). Alternating phases are achieved using a single-phase clock driving complementary switches.

In order to increase the I_{on}/I_{off} ratio of the switch transistors in the converter, the single-phase clock output of the oscillator is bootstrapped (boosted) to provide higher gate drive as shown in Supplementary Figure 2. In steady-state (for V_{in} = 150 mV), the boosted positive clock (for

driving NMOS switches) of 480 mV and boosted negative clock (for driving PMOS switches) of -200 mV. Steady-state I_{on}/I_{off} ratios of the devices are increased almost ten-fold from ~4600 to ~40,000, reducing conduction losses due to switch on-resistances. The bootstrap driver was critical in starting up the converter. At V_{in} = 4.5 kT/q, the I_{on}/I_{off} ratio was increased from ~30 to over 1200. We note that no startup frequency could be found without the use of the bootstrap circuit. An additional increase in transistor transconductance is achieved by using body-contacted devices and shorting the gate to the body in a dynamic-threshold configuration [S4].

The outputs of the ring oscillator loads are level shifted up to 1 V and brought off the chip (Supplementary Figure 3a). The widely used cross-coupled PMOS level shifter configuration is ineffective at level shifting the oscillator outputs (amplitudes can range from 220 mV – 550 mV up to 1 V). Instead, the level shifter configuration in Supplementary Figure 3b is employed, which exploits the strong dependence of transistor drive strength to V_T, particularly in the subthreshold region.

To maintain high efficiency at less than 200 nW input power, there is no output load regulation. Increasing output ripple and decreasing average output voltage with increasing load is observed. The non-zero output resistance, given by $R_o \sim 1/f_{clk}C_1$, can be minimized by increasing f_{clk}. The measured R_o is larger than this value because the output voltage droop decreases the actual switching frequency. Maximum efficiency is achieved when the switching frequency is reduced to the maximum tolerable output ripple. The resulting efficiency as a function of output load power (P_{load}) is shown in Supplementary Figure 4. The operating point of the converter in our system is noted in Fig. S11-4, yielding $\eta_{converter}$ of 59%.

Digital loading of the converter is accomplished using four inverter-based independent ring oscillators (two 11-stage and two 31-stage of varying V_T transistors), with varying oscillator frequencies attached to the output of the converter.

The die photo is shown in Supplementary Figure 5. The core design consumes .0015mm^2, including 0.00097mm^2 (60%) for the capacitors. Delivery of 100 nW load power at 265 mV output voltage yields a current density of 0.252mA/mm^2. This is directly attributable to the

relatively large 100pF capacitors used in the design to minimize both P_{cond} and P_{sw}, at the expense of converter area and reduced current density.

3. Switch (S) implementation discussion

The on-chip converter and associated clock drivers, comparators, and loads (which are all powered by the input power supplied by the biocell) can be run in three configurations based on the available power from the biocell.

Configuration 1. In this configuration, the biocell can supply sufficient power to overcome the input leakage into the converter to start-up and sustain steady-state, non-duty cycled operation. For this to be true, the available current of the input power source must be greater than 41.7 nA.

Supplementary Figure 6 shows the input leakage current into the converter at voltages below the start-up voltage (V_{start}) of 145 mV. This current includes the core converter and clock drivers and loads; the latter are attached to the output of the converter. At 145 mV, the input leakage is 13.5 nA. After start up, the additional DC average current drawn from the input (when only one of the ring-oscillator loads is on) to power both the converter (and clocks) as well as the load is 28.2 nA. The total current drawn from the supply is, therefore, 41.7 nA.

In this case, the harvesting capacitor C_{STOR} is not required and the converter will run continuously unless the input voltage droops below V_{min} = 110 mV.

Configuration 2. In this configuration, the biocell can supply sufficient power to overcome the leakage at voltages below the start-up voltage but has insufficient power to run the chip continuously. As a result, duty-cycled operation is required. This occurs with the input supply current is greater than 13.5 nA (the leakage at V_{start}) but below 41.7 nA, the required current for active operation.

In this case, the harvesting capacitor C_{STOR} is required. An explicit on-chip C_{STOR} of 100 pF combines with intrinsic capacitance of the biocell to provide a total effective capacitance of 250 pF. The explicit switch S and associated control circuitry can be omitted. Analysis of the efficiency can be carried out by assuming an “implicit” switch S, which is on when the input voltage exceeds V_{start} and is off when the input voltage is below V_{min}.

Configuration 3 (our system). In this configuration, the biocell cannot supply sufficient power to overcome chip leakage into the chip at voltages below V_{start}. Duty-cycled operation is also required in this case with an explicit switch which detaches the input source from the chip such that the input capacitor C_{STOR} can charge up to the necessary V_{start}.

For the presented system, the input current available from the biocell at 145 mV is only ~2.1 pA, necessitated this configuration. However, acknowledging the input current could be increased dramatically through the use of parallel bilayers, we neglected the power associated with the explicit switch S and comparator, which were implemented off-chip on the printed circuit board (PCB), in the efficiency calculation.

4. Effects of ion gradient on electrogenic pump behavior

We do not have measured data for impact of ionic concentration gradient changes for our ATPases; however, it was shown for similar ATPases (sodium-potassium pumps in guinea pig ventricular myocytes)[5], that a 10% reduction in short-circuit current is observed for a 20 mM change in the sodium concentration in the *cis* chamber (the initial concentration of sodium in the *cis* compartment was reduced from 50 mM to 30 mM). For our system, given the large compartment volumes and low overall ionic transport rate (e.g. low current), a sodium concentration change of 20 mM would take 1.3×10^4 years as shown below.

For 20 mM Na^+ concentration change in the *cis* chamber:

Starting concentration (C_i): 50 mM
Final concentration (C_f): 30 mM
Concentration change (ΔC): 20 mM
Volume (V): 1 ml
ATP turnover rate (k_{ATP}): 100 events/sec
Number of active ATPases (N): 10^5
Sodium ion transport per event: 3

\# of moles in concentration change (molarity is moles/L):

$\Delta C \times V = 2 \times 10^{-5}$ moles

\# of Na^+ ions in concentration change:

2×10^{-5} moles $\times N_A = 1.2 \times 10^{19}$ molecules, where N_A is Avogadro's number.

With 300 sodium ions transport per second per ion pump and 10^5 ion pumps, this concentration change will require:

$1.2 \times 10^{19} / (3 \times k_{ATP} \times N) = 1.2 \times 10^{19} / (3 \times 100 \times 10^5) =$

$1.2 \times 10^{19} / 3 \times 10^7 = 4 \times 10^{11}$ sec = **1.3×10^4 years**

to develop.

Extrapolated data for the impact on pump current due to potassium ionic concentration[S5], (reduction from 5 mM to 3.2 mM) under the same analysis indicate that it would take 1.7×10^3 years for an identical 10% reduction in pump current.

supplementary References

1 Wang, G. *et al.* in *Electron Devices Meeting, 2006. IEDM '06. International.* 1-4.

2 Chang, L. *et al.* in *VLSI Circuits (VLSIC), 2010 IEEE Symposium on.* 55-56.

3 Maksimovic, D. & Dhar, S. in *Power Electronics Specialists Conference, 1999. PESC 99. 30th Annual IEEE.* 54-59 vol.51.

4 Assaderaghi, F. *et al.* in *Electron Devices Meeting, 1994. IEDM '94. Technical Digest., International.* 809-812.

5 Glitsch, H. G. Electrophysiology of the sodium-potassium-ATPase in cardiac cells. *Physiol Rev* **81**, 1791-1826 (2001).
